# Development of a women’s empowerment index for Tanzania from the demographic and health surveys of 2004–05, 2010, and 2015–16

**DOI:** 10.1186/s12982-021-00103-6

**Published:** 2021-10-07

**Authors:** Andrew Evarist Mganga, Jenny Renju, Jim Todd, Michael Johnson Mahande, Seema Vyas

**Affiliations:** 1 Department of Epidemiology and Biostatistics, Institute of Public Health, Kilimanjaro Christian Medical University College, Moshi, Tanzania; 2 London School of Hygiene & Tropical Medicine, London, England; 3 National Institute of Medical Research, Mwanza, Tanzania

**Keywords:** Women’s empowerment, Index, Reliability, Construct validity

## Abstract

**Background:**

Women’s empowerment is a multidimensional construct which varies by context. These variations make it challenging to have a concrete definition that can be measured quantitatively. Having a standard composite measure of empowerment at the individual and country level would help to assess how countries are progressing in efforts to achieve gender equality (SDG 5), enable standardization across and within settings and guide the formulation of policies and interventions. The aim of this study was to develop a women’s empowerment index for Tanzania and to assess its evolution across three demographic and health surveys from 2004 to 2016.

**Results:**

Women’s empowerment in Tanzania was categorized into six distinct domains namely; attitudes towards violence, decision making, social independence, age at critical life events, access to healthcare, and property ownership. The internal reliability of this six-domain model was shown to be acceptable by a Cronbach’s α value of 0.658. The fit statistics of the root mean squared error of approximation (0.05), the comparative fit index (0.93), and the standardized root mean squared residual (0.04) indicated good internal validity. The structure of women’s empowerment was observed to have remained relatively constant across three Tanzanian demographic and health surveys.

**Conclusions:**

The use of factor analysis in this research has shown that women’s empowerment in Tanzania is a six-domain construct that has remained relatively constant over the past ten years. This could be a stepping stone to reducing ambiguity in conceptualizing and operationalizing empowerment and expanding its applications in empirical research to study different women related outcomes in Tanzania.

## Background

Empowerment has been defined as the expansion of assets and capabilities of poor people by removing formal and informal institutional barriers that prevent them from taking action to improve their wellbeing [1, 2]. Women’s empowerment is a complex and multidimensional issue which should consider the following: firstly, women are not a homogenous group and have varying characteristics within a population. Secondly, strategies for women’s empowerment should focus on policy actions that take effect at the household level. Thirdly, women’s empowerment at the systemic level should focus on transforming systems that support patriarchal structures [3].

The multidimensionality of women’s empowerment poses a significant challenge for policy and decision-makers, researchers and implementers [4, 5]. Composite indices such as the Gender-based Development Index (GDI), the Gender-based Empowerment Measure (GEM), and the Gender-Equality Index (GEI) have been designed to measure gender disparities in basic capabilities between men and women [6]. Yet these indices have been criticized for their methodological limitation of trading off data relevance and importance and geographical coverage [6]. Further the choice of indicators is constrained by what is available at the global level. Culminating in disadvantages for developing or low-income countries, where the indicators are not truly representative of the gender disparities that exist in these countries [7, 8].

Capturing the multi-dimensional structure of women’s empowerment quantitatively is challenging. In practice operationalizing empowerment has often used variables from discreet studies that are suitable to that context and population under study [9–17]. Culminating in no consistency in which context-specific variables can or should be used to define women’s empowerment in quantitative research.

In recent years multi-country studies across Sub-Saharan Africa (SSA) have tried to conceptualize women’s empowerment. The results suggested that women’s empowerment be operationalized into domains that include, but are not limited to, attitudes towards violence, social independence, decision making, and asset ownership [4, 5, 18, 19]. However, whilst these studies can make inferences across multiple SSA countries they lose the important nuance of context specificity within the countries themselves.

In order to understand an individual country’s progress to sustainable development goal-5 (SDG 5); which focuses on gender equality and empowerment and which acknowledges women as an important demographic in realizing development; context-specific measures are needed [20].

The hypothesis for this research was that a composite index to measure women’s empowerment in Tanzania will provide a holistic approach to assess individual and country level progress overtime. The aims of this study were to; develop a women’s empowerment index from the 2004–05, 2010, and 2015–16 Tanzanian demographic and health survey (TDHS); to test internal reliability and construct validity of these indices; and to assess how the structure of women’s empowerment in Tanzania has changed across the three demographic and health surveys.

## Methodology

### Study design, area and population

This analytical cross-sectional study used nationally representative data from the 2004–05, 2010, and 2015–16 TDHS which collect data from women aged 15–49 who were either usual residents or visitors in the household on the night before the survey, men aged 15–49 who were either usual residents or visitors in the household on the night before the survey, and children aged 6 to 59 month that had a guardian or parent’s consent [21–23]. The study population was all married or cohabiting women of reproductive age (15–49 years).

### Sample size and sampling

The DHS uses a sample that is generally representative at the national, residence, and regional level and the sampling technique is of a stratified, two-stage cluster design, where first enumeration areas are drawn from a census file and from each of these enumeration areas a sample of households is drawn [23]. Across all three surveys—used in this study—women who reported to have never been in union, divorced, or separated were excluded from analysis and this resulted in sample sizes of 8189, 6786, and 6310 from the 2004–05, 2010 and 2015–16 TDHS, respectively.

### Study variables used for developing the women’s empowerment index

Evidence of relevance from previous studies guided the selection of suitable variables from within the TDHS [2–5, 18, 19]. All levels of categorical variables were coded according to the direction of their influence on empowerment such that categories that reflected higher levels of empowerment had a higher ranking and those categories that were indicative of disempowerment had a lower ranking [18, 19]. The variables used in this study and how they were coded are described below.

The response for beating justified if the wife goes out without telling the husband/partner, beating justified if wife neglects the children, beating justified if wife burns the food, beating justified if wife argues with husband/partner, beating justified if the wife refuses to have sex with husband/partner were coded 0 for don’t know, 1 for yes, and 2 for no.

The response for the person who usually decides how to spend the woman’s earnings, the person who usually decides on the woman’s healthcare, the person who usually decides on large household purchases, the person who usually decides on what to do with husband/partner’s earnings, the person who usually decides on visits to family or relatives were coded 1 for husband/partner alone, 2 for woman alone, 3 for both woman and husband/partner.

The response for a woman’s educational attainment was coded 1 for no education, 2 for primary education, 3 for secondary education, and 4 for higher education.

The response for educational difference, which was measured in years, between a woman and their husband/partner was coded 1 for partner has more years of education, 2 for partner has the same years of education, and 3 for woman has more years of education.

The response for if the woman worked in the past year was coded 1 for no, 2 for worked in the year before, and 3 for currently working or on leave while those for earning from the woman’s work were coded 1 for payment in kind, 2 for cash and in kind, and 3 for cash only.

The response for earning ratio between a woman and her husband/partner was coded 1 for earning less than the husband/partner, 2 for about the same earning, and 3 for earning more than the husband/partner.

The responses for owning land alone or jointly with husband/partner and owning a house alone or jointly with husband/partner were coded 1 for does not own, 2 for lone ownership, 3 for joint ownership, and 4 for lone and joint ownership.

The responses for getting permission to go to health facility, getting money needed for treatment at health facility, distance to the health facility, and not wanting to go alone to the health facility were coded 1 for being a big problem and 2 for not being a big problem.

The variables Age at first birth, Age at first cohabitation, Frequency of watching television, and Frequency of reading newspaper or magazines were used as they were originally collected by the DHS.

### Statistical analysis

#### Factor analysis

Exploratory factor analysis (EFA) using variables presented in Table 1 was conducted to obtain the domains that represent women’s empowerment. In order to obtain meaningful domains, the suitability of the variables for exploratory factor analysis was tested using the Kaiser–Meyer–Olkin (KMO) test of sampling adequacy [24] where values greater than 0.70 were considered adequate. Domain retention was considered for those domains that together explained more than 50% of the total variance and variables were excluded if they had a loading of less than 30% or if they loaded on more than one domain [25]. Cronbach’s α was used to test the overall internal reliability of the index and that of individual domains [26, 27]. Here too, domains that had a Cronbach’s α value of less than 50% and variables that had a correlation that was not similar to the rest of the variables in the index were eliminated [28–31]. The final domain structure—the empowerment index—was obtained after performing an oblique Promax rotation.

The construct validity of these domains was checked through confirmatory factor analysis (CFA) [32] using the following fit statistics; the Root Mean Squared Error of approximation (RMSEA) and its respective 90% confidence intervals which represent parsimony of an index; the Comparative Fit Index (CFI) and Standardized Root Mean Squared Residual (SRMR) which represent relative and absolute fit of the index [31, 33]. The target for an index with good construct validity is a RMSEA with a value of less than 0.05, and where CFI and RMSR are close to 1 and 0 respectively [32].

A random half of the original data was obtained using the STATA command “random”, with respect to DHS clusters. All factor analyses were performed on one split-half sample and the other half was used to develop the index. All statistical analyses were performed using STATA version 14.2.

## Results

### The survey-based women’s empowerment index

#### Domains of the empowerment index and their reliability from the 2015–16 TDHS

**Fig. 1:**
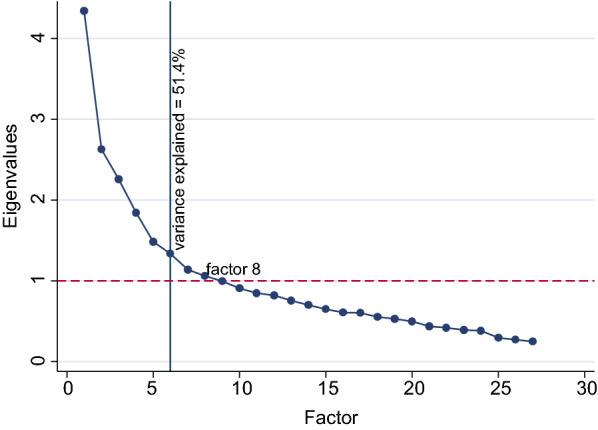
Scree plot of eigenvalues plotted against factors for the initial 27 variables used for exploratory factor analysis

**Fig. 2:**
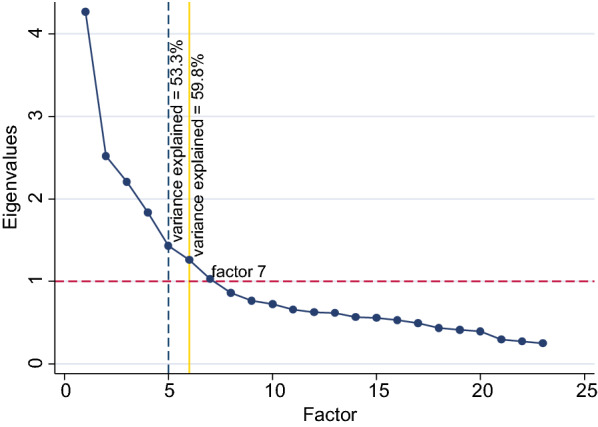
Scree plot of eigenvalues plotted against factors using measurement variables retained from the initial EFA

EFA utilizing 27 items representing empowerment in the 2015/16 TDHS revealed an 8-factor model for women’s empowerment. However, a variance of more than 59.8% was explained by a 6-factor structure (Fig. 1) composed of 23 items (Fig. 2) However, after rotation, the woman’s educational attainment variable loaded onto factors 3 and 4. A 5-factor model which contained 22 items and explained 53.3% of the variance (Fig. 2) had clean loading of variables across all factors and educational attainment was not a significant item in any of the factors.

Both the 6 and 5 factor models were compared in terms of reliability and construct validity given that they both explained a large amount of the variance in measuring empowerment and both models had adequate sampling adequacy (KMO = 0.789).

The 6-domains model of empowerment was composed of 23 items, five of these items loaded strongly on domain 1; five items loaded onto the second domain; five items loaded onto domain three; two items loaded onto the fourth domain; four items loaded onto the fifth; and the last domain was composed of two items. A description of the items, their respective domains and factor loadings are provided in Table 1.

**Table 1:** Results of oblique Promax rotation after EFA showing the construct structure of women’s empowerment in Tanzania

	Variables	Factor loading
Domain 1	Beating justified if the wife goes out without telling husband	0.836
	Beating justified if wife neglects the children	0.841
	Beating justified if wife argues with husband	0.893
	Beating justified if the wife refuses to have sex with the husband	0.818
	Beating justified if wife burns the food	0.712
Domain 2	The person who usually decides on respondent’s health care	0.733
	The person who usually decides on large household purchases	0.704
	The person who usually decides what to do with money husband earns	0.749
	The person who usually decides on visits to family or relatives	0.614
	The person who usually decides how to spend the respondent’s earnings	0.711
Domain 3	Owns a mobile telephone	0.682
	Frequency of watching television	0.717
	Frequency of reading newspaper or magazine	0.679
	Type of earnings from respondent’s work	0.389
	Educational attainment	0.542
Domain 4	Age of respondent at first birth	0.922
	Age at first cohabitation	0.939
Domain 5	Getting permission to get medical help	0.654
	Getting money needed for treatment	0.632
	Distance to a health facility	0.771
	Not wanting to go alone for medical help	0.758
Domain 6	Owns a house alone or jointly	0.891
	Owns land alone or jointly	0.874

#### Internal reliability and construct validity of the 5 and 6 domains models

**Fig. 3:**
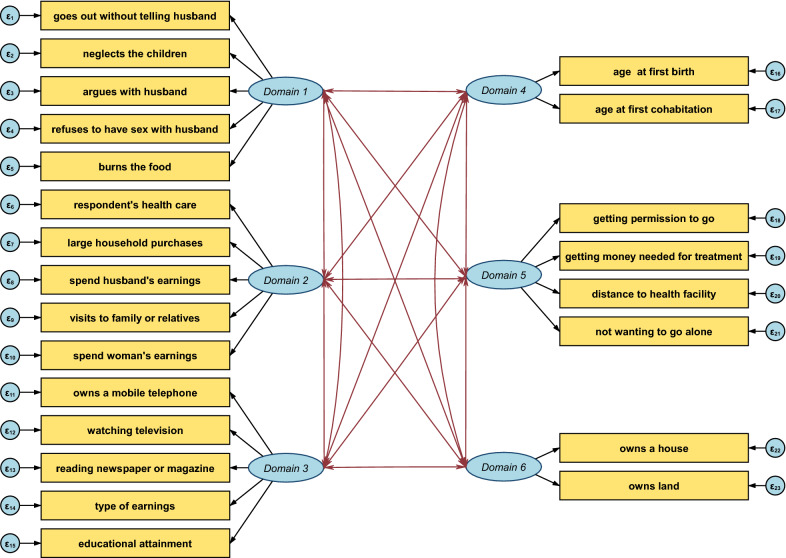
Path diagram used for assessing construct validation of the 6-domain model obtained from the 2015–16 TDHS using CFA

The 6-domains model had an overall, slightly better value of alpha (0.658) compared to the 5-domains model (0.653) (Table 2). The construct validity test illustrated that the 6-domains model was a better fit for measuring empowerment compared to the 5-domains model (Table 2). The value of RMSEA for the 6-domain model was at the 0.05 mark with a 90% confidence interval of 0.04 to 0.05 while the 5-domains model fell beyond the recommended threshold having an RMSEA value of 0.07 with a 90% confidence interval of 0.06 to 0.07 indicating that the 6-domain model was more parsimonious compared to the 5-domain model. In terms of absolute and relative fit, the 6-domain model had a CFI of 0.93 and an SRMR of 0.04 compared to 0.87 and 0.06 of the 5-domain model respectively, indicating that the 6-domain model was superior to the 5-domain model. The path diagram used to test construct validity for the 6-domain model is presented in Fig. 3.

**Table 2:** reliability of the items and domains of the 5 and 6-domain models

	Item-rest correlation	Average interitem covariance	Alpha	RMSEA (90% CI)	CFI	SRMR
Five- domain model						
Domain 1	0.574–0.772	0.133	0.875	–	–	–
Domain 2	0.189–0.684	0.941	0.593	–	–	–
Domain 3	0.456–0.548	0.308	0.749	–	–	–
Domain 4	0.392–0.533	0.067	0.663	–	–	–
Domain 5	–^a^	0.622	0.776	–	–	–
Overall reliability^b^	0.124–0.543	0.113	0.653	–	–	–
Construct validity	–	–	–	0.07 (0.06–0.07)	0.87	0.06
Six-domain model						
Domain 1	0.574–0.772	0.133	0.875	–	–	–
Domain 2	0.456–0.548	0.308	0.749	–	–	–
Domain 3	0.198–0.481	0.117	0.664	–	–	–
Domain 4	–^a^	9.579	0.816	–	–	–
Domain 5	0.392–0.533	0.067	0.663	–	–	–
Domain 6	–^a^	0.622	0.776	–	–	–
Overall reliability^c^	0.103–0.539	0.128	0.658	–	–	–
Construct validity	–	–	–	0.05 (0.04–0.05)	0.93	0.04

^a^Not available for domains with only two measured variables

^b^For all 22 items in the 5-domain model

^c^For all the 23 items in the 6-domain model

From this point forward the nomenclature of these domains will be similar to those from Ewerling [4], Asaolu [19], and Miedema [18]. Therefore, these domains will be named; attitudes towards violence, decision making, social independence, age at critical life events, access to healthcare, and property ownership.

#### Comparing the structures of empowerment across surveys in Tanzania

The structure of women’s empowerment has remained relatively constant over a time span of 10 years—from the 2004–05 survey to the 2015–16 survey (Table 3). Slight variations in the number of items at each survey year was due to the elimination of old questions and the inclusion of new ones with progressively subsequent surveys. Notably the removal of variables such as final say on making household purchases for daily needs and food to be cooked each day from the decision-making domain and having to take transport to get medical care from the access to healthcare domain from the 2010 and 2015–16 surveys. And the introduction of whether a woman owns a mobile phone in the 2015–16 survey which loaded onto the social independence domain. The structure of the attitudes towards violence domain remained the same over the ten year period that the surveys represent and was measured by the same five questions on justifying wife-beating when “she goes out without telling her husband/partner”, “neglects the children”, “argues with her husband/partner”, “refuses to have sex with her husband/partner”, and “burns the food”. The decision-making domain remained stable despite the removal of variables mentioned earlier and the introduction of the measure of “who decides what to do with the husband/partner’s earnings” in the 2015–16 survey. The social independence and age at critical life events domains had some fluctuations in their structures in terms of “educational attainment” where in 2004–05 it was shown that educational attainment loaded in the latter domain, the opposite was observed in the subsequent surveys where it loaded onto the former domain. The social independence domain also included “use of mobile phone” which was first introduced in the 2015–16 survey. The property ownership domain consisted of variables that have been measured the same way across all surveys, “ownership of a house” and “ownership of land”.

**Table 3:** comparison of empowerment domains and their structure across survey years following exploratory factor analysis

Domain	Measured variables	Factor loading by survey year		
		2004–05	2010	2015–16
Attitudes towards violence	Beating justified if the wife goes out without telling husband	0.7946	0.8325	0.8358
	Beating justified if the wife neglects the children	0.8241	0.8628	0.8407
	Beating justified if the wife argues with husband	0.8084	0.8730	0.8932
	Beating justified if the wife refuses to have sex with the husband	0.7631	0.8334	0.8181
	Beating justified if the wife burns the food	0.6672	0.6783	0.7115
Decision making	The person who usually decides on the respondent’s health care	0.7210	0.7771	0.7325
	The person who usually decides on large household purchases	0.7216	0.7891	0.7039
	The person who usually decides what to do with money husband earns	–	–	0.7493
	The person who usually decides on visits to family or relatives	0.7610	0.7953	0.6141
	The person who usually decides how to spend the respondent’s earnings	0.4711	0.5504	0.7105
	Final say on making household purchases for daily needs	0.7918	–	–
	Final say on food to be cooked each day	0.5494	–	–
Social independence	Owns a mobile telephone	–	–	0.6817
	Frequency of watching television	0.6833	0.7342	0.7173
	Frequency of reading newspaper or magazine	0.6909	0.5747	0.6793
	Type of earnings from respondent’s work	0.6487	0.6868	0.3888
	Educational attainment	–	0.6248	0.5422
	Work for family, others, self	0.5178	–	–
Age at critical life events	Age of respondent at first birth	0.9256	0.9184	0.9216
	Age at first cohabitation	0.9165	0.9244	0.9387
	Educational attainment	0.4190	–	–
Access to healthcare	Getting permission to go	–	0.7281	0.6541
	Getting money needed for treatment	0.5261	0.6146	0.6319
	Distance to health facility	0.8249	0.7178	0.7706
	Not wanting to go alone	0.6701	0.8204	0.7581
	Having to take transport	0.8421	–	–
Property ownership	Owns a house alone or jointly	0.8183	0.8407	0.8911
	Owns land alone or jointly	0.8312	0.8414	0.8737

**Fig. 4:**
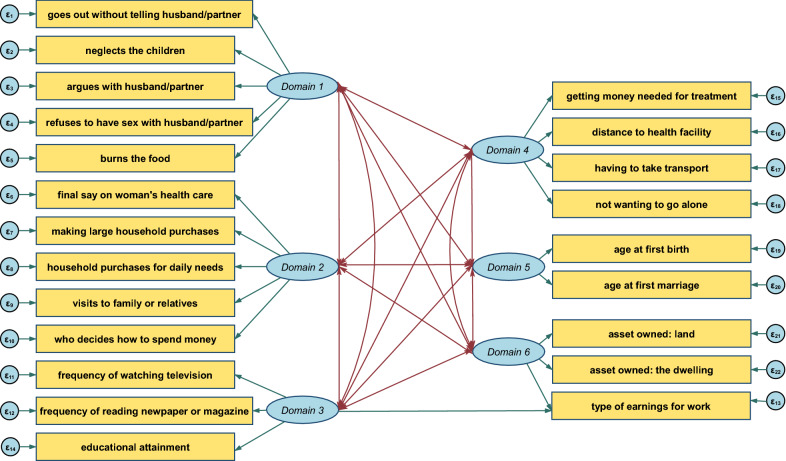
Path diagram for testing construct validity of a women’s empowerment index from the 2004–05 TDHS using CFA

**Fig. 5:**
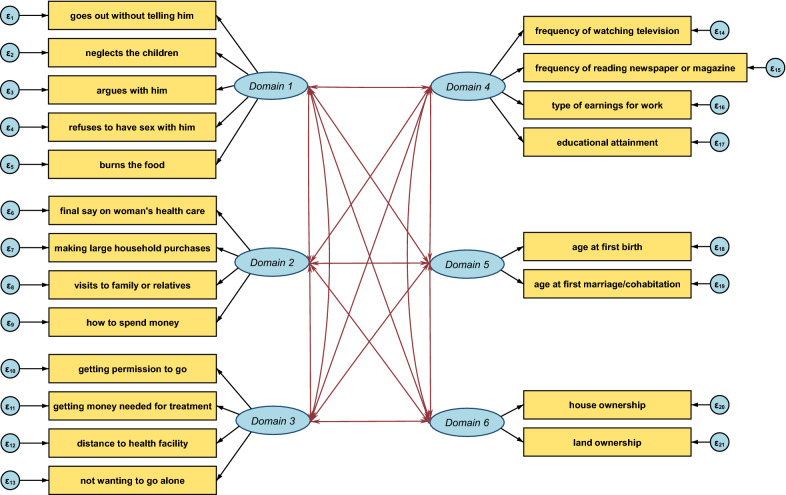
Path diagram for testing construct validity of a women’s empowerment index from the 2010 TDHS using CFA

The empowerment indices of 2004–05 and 2010 both had a good statistical fit with acceptable reliability (α > 0.6) and construct validity. The path diagrams for these indices are provided in Figs. 4 and 5 and their respective fit statistics and measure of internal reliability are provided in Table 4.

**Table.4:** Internal reliability and construct validity of the indices obtained from the 2004–05 and 2010 TDHS

	2004–05 survey	2010 survey
Cronbach’s α	0.619	0.614
RMSEA (90% CI)	0.051 (0.046–0.056)	0.045 (0.041–0.049)
CFI	0.927	0.935
SRMR	0.048	0.038

## Discussion

This study aimed to develop a women’s empowerment index for Tanzania and assess how the structure of this index has changed across three demographic and health surveys. By using exploratory and confirmatory factor analysis this study has developed a reliable and valid six-domain construct of women’s empowerment in Tanzania which reduces ambiguity in quantitatively conceptualizing and operationalizing empowerment. The six domains are attitudes towards violence, decision making, social independence, age at critical life events, access to healthcare, and property ownership. This study has also shown that the structure of women’s empowerment in Tanzania has remained relatively static across three demographic and health surveys from 2004 to 05 to 2015–16.

Previous studies across sub-Saharan Africa have conceptualized women’s empowerment using just four domains [4, 5, 18, 19]. By conceptualizing across countries these studies reduce their scope to capture the multi-dimensionality of the construct. This study found that a valid and reliable Tanzanian specific index for women’s empowerment across a 10-year period should be composed of six domains measured by 23 items. Limiting the measure to just 4 domains is likely to underestimate the multidimensionality of women’s empowerment in Tanzania.

There were three domains from this study namely, attitudes towards violence, decision making, and access to healthcare that were similar to those identified by Asaolu [19], Miedema [18], and Ewerling [4]. However, there appeared to be a marked difference in the social independence domain. In Tanzania social independence was comprised of educational attainment, owning a mobile phone, frequency of watching television and reading newspapers or magazines, and the type of earnings from the woman’s work while the variables age at first birth and age at first cohabitation were represented in a separate domain. Some variables such as frequency of watching television and owning a mobile phone are not collected or available in the DHS surveys for all countries in SSA, it is therefore possible that this led Ewerling and colleagues [4] to include the following variables in the social independence domain: frequency of reading newspapers, woman’s educational attainment, age at first cohabitation, and age at first birth, in order to accommodate data from countries that missed one or more elements of the domain. For the same reason, property ownership emerged as an important domain for women’s empowerment in Tanzania whereas in all other SSA studies this domain was missing. These points illustrate that whilst there is utility in a cross-country model, the tradeoff is to miss key measurement variables that can be used in developing a country-specific model of women’s empowerment.

The attitudes towards violence domain emerged as the most important domain for women’s empowerment in Tanzania. The justification of violence against women is accepted by a substantial proportion of men and women in Africa [34], even in Tanzania the proportion of women accepting “wife-beating” remains to be high (58%) as reported by the 2015–16 DHS [35]. Indeed attitudes towards violence have been highlighted in various literature as important factors for women’s experience of IPV, where greater acceptance of such attitudes is positively correlated with reports of IPV [36, 37].

Kabeer’s definition of empowerment, includes decision making as part of a woman’s agency—specifically her “ability to define goals and act upon them” [2]. Women’s decision making power, either alone or jointly, has been shown to be positively associated with outcomes such as improved dietary diversity, contraceptive uptake, and utilization of maternal healthcare services and negatively associated with IPV [9, 10, 14, 17, 38]. In this study, decision making emerged as the second most important domain for women’s empowerment in Tanzania.

Access to healthcare was another important domain in defining women’s empowerment in Tanzania. Asaolu and colleagues point out that the variables in the access to healthcare domain—permission, money, distance—represent whether women have access to make beneficial health choices and this is important to empowerment [19]. In the financial year of 2016/17 the WHO reported the average Tanzanian household had an out-of-pocket expenditure on health of 21.89% which was higher than that of Rwanda(6.38%), Mozambique (7.67%), Zambia (12.12%), and Malawi (11.39%) [39, 40]. Given the reduced national expenditure on health from TZS 1988.2 billion in 2016/17 to TZS 1731.8 billion in 2018/19 [41] it is likely that high levels of out-of-pocket expenditure will remain and continue to perversely impact on a women’s ability to access quality and timely healthcare. .

Property ownership has been used in literature as one of the factors for defining empowerment [42–44]. However, cross-sectional studies in Tanzania and South Africa have demonstrated that women who reported owning property in the form of land or a house also had higher odds of experiencing IPV when compared to those who did not [42, 43]. Such contradictions suggest that the relationship between property ownership and IPV is not so straight forward, further cautioning that the effect of property ownership might vary from one setting to another and by context [45, 46].

The major strength of this study is the conceptualization of a holistic, valid and reliable approach to the operationalization of women’s empowerment for quantitative research in Tanzania. The internal validity of the data used to develop the empowerment index is guaranteed by the multi-stage sampling strategy used by the demographic and health survey which ensured that the data was sampled with probability proportional to enumeration area size and was adequately representative of the country’s population. Further validity of the data was demonstrated by the results of confirmatory factor analysis which showed optimal performance of the index across three separate surveys using the measures of parsimony (RMSEA) and fit (CFI & SRMR). The data was also shown to be adequately reliable for the construction of the empowerment index by the use of Cronbach’s α where the cut-off for reliability was set at 0.5 and across all survey, the value of α was shown to be above 0.6. Another strength is that the use of a nationally representative dataset such as the TDHS has allowed this study to flesh out the meaning of women’s empowerment in the Tanzanian context.

However, this study does have some limitations that should be considered when interpreting the findings. Firstly, the variables used to develop the index are self-reports of women and could be affected by a social-desirability bias. Secondly, the variables that are used to measure women’s empowerment change with time, they might be important in a context when they are at the incipient stage but become less important or useful when they become normative. Therefore, there might be a need to revise the composition of the empowerment index from this study to ensure that it remains up to date and relevant to Tanzania. Finally, because most of the variables used to develop the empowerment index were obtained from the TDHS, there might be some important information that is pertinent to empowerment to a married or cohabiting Tanzanian woman such as, financial and market autonomy or efficient utilization of assets, that is not captured in the survey and hence could not be used for analysis.

## Conclusions

This research was designed to conceptualize a country-specific index for women’s empowerment in Tanzania. This research found that women’s empowerment in Tanzania is a six-domain construct composed of attitudes towards violence, decision making, social independence, age at critical life events, access to healthcare, and property ownership. This finding will help reduce ambiguity in conceptualizing and operationalizing empowerment in empirical research within the Tanzanian setting and provide a holistic approach to studying outcomes such as partner violence and can be expanded to other areas such as maternal, newborn and child health, child spacing, and fertility. Although this research has shown that the structure of empowerment has not changed over a 10-year period there is still a need to update the empowerment index since population and behaviours change.

## Data Availability

The datasets generated and/or analyzed during the current study are available in the demographic and health survey program repository, “https://dhsprogram.com/what-we-do/survey/survey-display-485.cfm”.

## Abbreviations

CFA: Confirmatory factor analysis
CFI: Comparative fit index
DHS: Demographic and Health Survey
EFA: Exploratory factor analysis
GDI: Gender Development Index
GEI: Gender Equality Index
GEM: Gender-based empowerment measure
KMO: Kaiser–Mayer Olkin
RMSEA: Root mean squared error of approximation
SRMR: Standardized root mean squared residual
TDHS: Tanzania Demographic and Health Survey
